# Prefrontal Theta Oscillations Promote Selective Encoding of Behaviorally Relevant Events

**DOI:** 10.1523/ENEURO.0407-18.2018

**Published:** 2018-01-10

**Authors:** Justin Jarovi, Julien Volle, Xiaotian Yu, Lisa Guan, Kaori Takehara-Nishiuchi

**Affiliations:** 1 Department of Cell and Systems Biology; 2 Department of Psychology; 3 Neuroscience Program, University of Toronto, Toronto M5S 3G3, Canada

**Keywords:** associative learning, medial prefrontal cortex, memory enhancement, theta oscillations

## Abstract

The ability to capture the most relevant information from everyday experiences without constantly learning unimportant details is vital to survival and mental health. While decreased activity of the medial prefrontal cortex (mPFC) is associated with failed or inflexible encoding of relevant events in the hippocampus, mechanisms used by the mPFC to discern behavioral relevance of events are not clear. To address this question, we chemogenetically activated excitatory neurons in the mPFC of male rats and examined its impact on local network activity and differential associative learning dependent on the hippocampus. Rats were exposed to two neutral stimuli in two environments whose contingency with an aversive stimulus changed systematically across days. Over 2 weeks of differential and reversal learning, theta band activity began to ramp up toward the expected onset of the aversive stimulus, and this ramping activity tracked the subsequent shift of the set (stimulus modality to environment) predictive of the aversive stimulus. With chemogenetic mPFC activation, the ramping activity emerged within a few sessions of differential learning, which paralleled faster learning and stronger correlations between the ramping activity and conditioned responses. Chemogenetic mPFC activity, however, did not affect the adjustment of ramping activity or behavior during reversal learning or set-shifting, suggesting that the faster learning was not because of a general enhancement of attention, sensory, or motor processing. Thus, the dynamics of the mPFC network activation during events provide a relevance-signaling mechanism through which the mPFC exerts executive control over the encoding of those events in the hippocampus.

## Significance Statement

Although we now have a fair understanding of memory encoding, the real-time processes through which the brain controls the strength of memory encoding remain mostly unknown. Developing an understanding of these memory regulation processes is important because it informs a new strategy for memory enhancement, which is critically needed for the fast-growing elderly population facing memory handicaps. We discovered that activating one region of the brain, the medial prefrontal cortex (mPFC) facilitated rats to form memories of important events without increasing the learning of unimportant details. The memory enhancement was associated with improved responses of mPFC network to the important events. Manipulations of the mPFC, therefore, allow for tapping into intrinsic memory encoding processes to enhance event memories.

## Introduction

The hippocampus and surrounding rhinal cortices are essential for the rapid encoding of complex spatial and temporal sequences of events that we encounter during everyday life (Squire, 1992; Eichenbaum, 2000). These early plastic changes within the hippocampal circuits subsequently induce gradual reorganization of neocortical circuits through which memory traces become consolidated (Squire, 1992; Nadel and Moscovitch, 1997; Frankland and Bontempi, 2005). Several lines of evidence, however, suggest that the strong activation of the neocortex, in particular, the prefrontal cortex (PFC), during events is also essential for successful memory encoding. In the human brain, the hippocampus and prefrontal cortex are coactivated during memory encoding, and the ability to later remember events is predicted by the magnitude of prefrontal activation during encoding (Brewer et al., 1998; Wagner et al., 1998; Kao et al., 2005; Kirchhoff et al., 2000; Otten et al., 2002). In rodents, transcriptional, structural, and functional remodeling occurs in the medial PFC (mPFC; Bero et al., 2014; Kitamura et al., 2017) and orbitofrontal cortex (OFC; Lesburguères et al., 2011) at the time of encoding, and the activation of the mPFC is necessary for gene expression leading to synaptic modifications in hippocampal neurons (Bero et al., 2014). These findings raise the possibility that the PFC controls the activity of the hippocampus during learning, thereby determining whether certain information is made into long-term storage.

The top-down control that the PFC exerts over hippocampal encoding processes bears a resemblance to its established role in executive control, which relies on flexible, context-specific use of goals and expectations (Miller and Cohen, 2001; Euston et al., 2012). In well-trained animals, a sizable proportion of neurons in the mPFC and OFC respond to environmental stimuli that predict upcoming rewards (Schoenbaum and Eichenbaum, 1995; Pratt and Mizumori, 2001; Hosokawa et al., 2007; Morrison and Salzman, 2009; Insel and Barnes, 2015) or aversive stimuli (Baeg et al., 2001; Gilmartin and McEchron, 2005; Hosokawa et al., 2007; Takehara-Nishiuchi and McNaughton, 2008; Morrison and Salzman, 2009). Some have proposed that the observed neural selectivity for behaviorally relevant stimuli may underlie the role of these regions in memory retrieval and memory-guided decision-making: the mPFC represents rules (a learned mapping of stimuli and adaptive behavior; Euston et al., 2012; Eichenbaum, 2017), whereas the OFC uses these rules according to the task state (the current location in a task; Wilson et al., 2014; Wikenheiser and Schoenbaum, 2016). A question remains open as to whether the same neural selectivity underlies the emeging role of the PFC in memory encoding.

To address this point, we used chemogenetic approaches to artificially generate the mPFC network state associated with encoding success and examined its impact on behavior in trace eyeblink conditioning. In this hippocampus-dependent memory paradigm, animals associate a neutral stimulus with a mildly aversive stimulus to the eyelid over a short temporal gap (Woodruff-Pak and Disterhoft, 2008; Takehara-Nishiuchi, 2018). In the present study, we used two neutral stimuli and systematically changed their contingency with the aversive stimulus across days. To determine the effect of the manipulation on the mPFC network responses to the stimuli, we also recorded local field potentials (LFPs) from the mPFC during the conditioning. We found that the mPFC network responses differentiated the behaviorally relevant stimulus (i.e., paired with the aversive stimulus) from the irrelevant stimulus (i.e., presented alone). Chemogenetic mPFC activation promoted the development of the selective network responses and in turn facilitated the initial formation of differential stimulus associations. It, however, did not affect subsequent adjustment of behavior and the network responses according to the changing stimulus contingency.

## Materials and Methods

### Subjects

Twenty-four male Long–Evans rats (Charles River Laboratories), 70 d old on arrival, were individually housed in transparent plastic cages in a home colony room and maintained on a 12 h reverse light/dark cycle (dark from 10:00–22:00) with *ad libitum* access to food and water. Their weight ∼1 week after their arrival ranged from 350 to 425 g. Behavioral experiments were performed during the dark cycle. Five rats were excluded because of insufficient expression of the transgene, leaving 19 rats for the analysis of behavioral data. Two additional rats were excluded because of misplaced electrodes, leaving 17 rats for the analysis of LFP data. One of these rats lost the head implant during the third stage of conditioning (see Behavioral analyses section). This rat was removed from behavioral and LFP analyses during the set-shift stage. Among the 19 rats included in the behavioral analysis, 10 rats were used for the analysis of c-Fos expression. In addition to the 24 rats that were purchased for this study, we reanalyzed c-Fos expression data collected from 15 rats in our previous study (Volle et al., 2016). All procedures were in accordance with the National Institutes of Health *Guide for the Care and Use of Laboratory Animals* (Publication No. 85-23, revised 1985), the Canadian Council on Animal’s Care, APA ethical standard, and approved by the University of Toronto Animal Care Committee.

### Viral vectors

Recombinant adeno-associated virus (AAV) vectors encoding the Designer Receptors Exclusively Activated by Designer Drug (DREADD; Armbruster et al., 2007; Rogan and Roth, 2011) was a gift from Bryan Roth; University of North Carolina School of Medicine (Addgene viral prep #50476-AAV8). All rats received intracranial injection of pAAV8-CaMKIIa-hM3D(Gq)-mCherry, and the titer was 3 × 10^12^ vg/ml.

### Surgery

Rats underwent two surgery protocols: they first received the viral vector (1) and then after at least a 5 week incubation period were implanted with LFP and electromyogram (EMG) electrodes (2).

***Viral vector surgery (1).***One week after their arrival at the facility, rats were anesthetized (1–2% isoflurane by volume in oxygen at a flow rate of 0.8 L/min; Fresenius Kabi) and placed in a stereotaxic frame. Skin was retracted and holes drilled in the skull bilaterally above the prelimbic region of the mPFC [anteroposterior (AP) = +3.2, mediolateral (ML) = ±0.5 from bregma; Paxinos and Watson, 2007)]. A 30 G stainless steel cannula was connected to a microsyringe (Hamilton) via polyethylene tubing and lowered to 3.5 mm below bregma. The viral vector (pAAV8-CaMKIIa-hM3D(Gq)-mCherry; 0.5 μl/side) was injected at a rate of 0.1 μl/min. The cannula was left in place for an additional 10 min following the microinjection to ensure the diffusion of the vector. The cannula was then slowly retracted, and the incision was sutured. The rats were treated with analgesic (carprofen, 5 mg/kg, s.c.) for 48 h after surgery and left in their home cage for 5 weeks.

***Electrodes surgery (2).***Bipolar LFP electrodes were fabricated by inserting two short pieces of stainless steel wire (791000, A-M Systems) into a 25 G stainless steel cannula (distance between electrodes tips was 0.7 mm). As described in the previous section (1), rats were anesthetized and placed in a stereotaxic frame. After craniotomy and removal of dura, an electrode was implanted in the prelimbic region of mPFC (AP = +3.2, ML = +0.6, DV = −3.9 mm from bregma) contralateral (right) to the conditioned eye. In addition, four Teflon-coated stainless-steel wires (791000, A-M Systems) were implanted subcutaneously in the upper left orbicularis oculi (eyelid muscle) to record EMG activity and deliver a periorbital shock. All electrode wires were attached to a connector cap (Ginder Scientific), and the connector cap was secured to the skull with stainless steel screws and dental acrylic resin. The rats were given an analgesic treatment [see Viral vector surgery (1)] and at least 7 d to recover from surgery before beginning daily conditioning sessions.

### Histology

On the completion of behavioral testing, brain tissue was collected to conduct three experiments to address the following points: Experiment A, the location and spread of viral vector infection; Experiment B, the location of LFP electrodes; and Experiment C, the *ex vivo* validation of the cumulative impact of clozapine-*N*-oxide (CNO) treatment and trace eyeblink conditioning on the activity of mPFC neurons.

#### Tissue collection

Rats were subcutaneously injected with either CNO (0.1 mg/kg body weight) or 0.9% physiologic saline solution before the start of conditioning, using the same drug assignment the rats received during conditioning sessions. Five rats were injected with saline and five rats were injected with CNO. Following a 30 min wait, the rats underwent conditioning for 10 min. After conditioning, 50 min later, rats were deeply anesthetized with an excess amount of sodium pentobarbital (102 mg/kg; Euthansol, Merck), and the tips of the LFP electrodes were marked by electrolytic lesions (20 µA for 45 s, positive to electrode, negative to animal ground). They were perfused transcardially with chilled 0.9% physiologic saline solution, followed by chilled 4% paraformaldehyde (PFA). Brains were extracted and left submerged within 4% PFA at 4°C overnight, followed by PBS-30% sucrose solution (PBS-Suc) for 48 h. Coronal brain slices (40 μm) across the entire anterior–posterior extent of the medial prefrontal cortex were collected using a cryostat (CM3050S, Leica Biosystems). Every first, and second tissue section was stored in a separate tube filled with tissue storage buffer (TSB; PBS with sucrose and ethylene glycol solution), whereas every third tissue section was stored in PBS, all tubes aside from those stored in only PBS were then stored at −20°C. Each tube was used for Experiments A, B, and C.

#### Location and extent of viral infection

For Experiment A, sections were mounted on a glass slide and coverslipped with ProLong Diamond antifade mounting medium containing DAPI (ThermoFisher Scientific). Endogenous mCherry signal was used to determine the placement and extent of viral infection for each rat. Rats that received pAAV8-CaMKIIa-hM3D(Gq)-mCherry were included in subsequent analyses if robust mCherry expression was observed in the prelimbic region in at least one hemisphere.

#### Location of LFP electrodes

For Experiment B, tissue was mounted on a glass slide and stained with cresyl violet. It was subsequently examined under a light microscope, and the locations of the electrode tips were drawn on to templates from the stereotaxic atlas of the rat brain (Paxinos and Watson, 2007). All data from the two rats whose electrodes were not located in the prelimbic region were omitted from the analyses (1 saline-treated and 1 CNO-treated rat).

#### Immunostaining

For Experiment C, sections were removed from TSB, washed in PBS, and submerged in 20% goat serum (ab156046, Abcam) for 2 h at room temperature. The sections were then washed three times with PBS-0.1% Triton X-100 Detergent (PBS-T). Then, they were incubated with a primary antibody against c-Fos (rabbit, #2250, 1:400; Cell Signaling Technology) at 4°C overnight. Subsequently, the sections were washed three times using PBS-T and then incubated in a secondary anti-rabbit IgG antibody conjugated with AlexaFluor 488 (Goat, #111-545-003, 1:400; Jackson ImmunoResearch) for 2 h at room temperature. The sections were washed three times with PBS, and were mounted on glass slides and sealed with coverslips and ProLong Diamond antifade mounting medium containing DAPI, as in Experiment A.

#### Image acquisition

Images were collected from the immunostained tissue using an upright fluorescence microscope (Axio Imager, Z1, Zeiss) with a 20× objective. Areas of analysis consisted of a 0.35 × 0.35 mm region on the *x*–*y* scale. A section with the track of cannula used for the viral vector injection was identified. Then, an image was taken from an area adjacent to the injection track. Three images were collected from the prelimbic region from each rat.

#### Cell counting

Collected images were manually analyzed by two experimenters who were blind to the treatment condition with the help of FIJI (ImageJ) software. In each image, the number of DAPI-positive cells was marked and counted. Small, bright uniformly DAPI stained nuclei from putative glial cells were not included in the DAPI counts. The number of DAPI-positive cells that were also positive for c-Fos- or hM3Dq (detected as mCherry signal) were counted. The raw count was converted to a ratio for each image. The proportion of c-Fos expressing cells [P(c-Fos)] was calculated as the number of c-Fos expressing cells that were also DAPI-positive divided by the total number of DAPI-positive cells. In a separate cohort of six animals, we tested the possibility of a nonselective effect of CNO on c-Fos expression. Rats were transfected in the same manner previously described with either rAAV2/8-CamKIIa- eGFP or rAAV2/8-CamKIIa-hM3D (Gq)-mCherry (purchased from the vector core at the University of North Carolina) and randomly assigned to one of three groups: hM3Dq-Sal, hM3Dq-CNO, GFP-CNO. Animals were injected with either CNO or saline, immediately placed back into their home cages, and perfused 90 min after. P(c-Fos) in the prelimbic region of the mPFC was calculated in the same manner as our other experiment.

### Behavioral analyses

#### Drug assignments

At least 6 weeks after the intracranial injection of pAAV8-CaMKIIa-hM3D(Gq)-mCherry, the rats were randomly assigned to receive subcutaneous injections of either CNO (Tocris Bioscience, catalog #4936) dissolved in 0.9% saline solution (0.1 mg/kg body weight, *n* = 10) or saline solution (*n* = 9) 30 min before daily conditioning sessions (starting from the second adaptation session). All rats received the same treatment for the entirety of the experiment.

#### Trace eyeblink conditioning

All rats were trained in trace eyeblink conditioning for 23 d each of which consisted of two conditioning epochs in two different conditioning environments (Contexts 1 and 2) in a sound and light-attenuating chamber. Context 1 consisted of a transparent, cylindrical, plastic container with P80 grit sandpaper covering the floor. The floor was kept at 15°C through the use of a hot/cold compress pack. Context 2 consisted of a striped black and white walled, rectangular, plastic container with paper towel covering the floor. The floor was kept at 25°C through the use of the hot/cold compress pack. Rats were counterbalanced such that half the animals underwent the first conditioning epoch in Context 1 and the other half in Context 2. In the second epoch, animals were placed into the opposite context from the first epoch.

For the first two sessions, rats were placed in the conditioning chamber for 50 min without any stimulus presentations to habituate them to the procedures and chamber. Starting on the third session, rats underwent 7 d of conditioning in each of the three learning stages of differential trace eyeblink conditioning. Rats were presented with the conditioned stimulus (CS), which was presented for 100 ms and consisted of an auditory stimulus (85 dB, 2.5 kHz pure tone) or a visual stimulus (white LED light blinking at 50 Hz). The unconditioned stimulus (US) was a 100 ms mild electrical shock to the eyelid (100 Hz square pulse). The auditory CS was delivered through a speaker in the chamber ceiling, and the visual CS was delivered through an LED light placed on the wall of the chamber. The US was delivered to the eyelid with a stimulus isolator (ISO-Flex, AMPI). The shock level was initiated at 0.3 mA and was adjusted individually and daily for each rat to induce an optimal unconditioned response (an eyeblink/head-turn response), which was monitored through web cameras mounted inside the chambers. Stimulus delivery was controlled by a RZ-5 recording system (Tucker-Davis Technologies) with a microcontroller (Arduino Mega).

Daily recording sessions consisted of two epochs of conditioning, each with 100 trials, separated by a 10 min rest period. The intertrial intervals were pseudorandomized between 20 and 40 s with a mean of 30 s. Each epoch included 50 presentations of the auditory CS and 50 presentations of the visual CS, pseudorandomized over the course of the 50 min recording. During the first stage (Differential Learning, Days 3–9), one of the CS (CS1; e.g., auditory CS) was paired with the US with a 500 ms stimulus-free interval between them, whereas the other type of CS (CS2; e.g., visual CS) was presented alone in both Contexts 1 and 2. During the second stage (Reversal Learning, Days 10–16), CS–US contingency was reversed in such a manner that rats received pairings of CS2 and US, whereas CS1 was presented alone, in both Contexts 1 and 2. During the third stage (Set-Shift, Days 17–23), both CS1 and CS2 were paired with the US in Context 1, whereas both CS were presented alone in Context 2.

During conditioning, eyeblink responses were monitored by recording EMG activity from the left upper orbicularis oculi muscle through two surgically implanted stainless steel wires. EMG activity was bandpass filtered between 0.3 and 3.0 kHz, digitized at 6102 Hz, and stored using a RZ-5 recording system (Tucker-Davis Technologies).

#### EMG analysis

All analyses were performed using custom code written in MATLAB (MathWorks) with the same procedure as that used in our previous studies (Volle et al., 2016; Morrissey et al., 2017). For each session per animal, the amplitude of the EMG signal during each 1 ms of time was calculated by subtracting the minimum signal from the maximum signal during that 1 ms window. EMG amplitude was averaged during a 300 ms window before CS presentation in each trial. The baseline was set as the median of averaged EMG amplitude plus 1 SD. EMG activity above the threshold was averaged together during the 300 ms pre-CS period (pre-value) and during a 200 ms window before US onset (CR value). The CR value was designed to capture the adaptive, anticipatory blinking responses that occur immediately before US onset. A trial was defined as a CR trial if the CR value was at least five times that of the pre-value. In some trials, the pre-value exceeded 10 times the threshold because the rats engaged in grooming, teeth grinding, or climbing immediately before CS onset (Morrissey et al., 2017). These trials were classified as “hyperactive” trials and discarded. The percentage of conditioned responses (CR%) for each animal in a given session was the ratio of CR trials relative to the total number of valid trials. The significance of differentiation of CR% between two CS types was tested with random permutation tests. In each rat, in each session, the differential index (*DI_CR*) was calculated as follows: *DI_CR* = abs(*CR1%* − *CR2%*)/(*CR1%* + *CR2%*), where *CR1%* and *CR2%* are the percentage of CR trials in CS1 and CS2 trials, respectively. Then, trial types were randomly reassigned to each trial, and *DI_CR* was calculated. This procedure was repeated 1000 times to construct sampling distributions. The *DI_CR* with the real trial label was considered significant when it fell in the 0.05% of upper tail of its corresponding distribution. The number of sessions needed for *DI_CR* to reach the significance was counted in each rat. To examine the temporal pattern of EMG activity in each learning stage, EMG amplitude was averaged across all valid trials in the final session of each learning stage, then across animals in each group. EMG amplitude was divided by the averaged amplitude during a 300 ms window before the CS onset for normalization.

### Electrophysiological analyses

General procedures for LFP recording and analyses are the same as those used in our previous study (Volle et al., 2016). All analyses were conducted with custom codes written in MATLAB (MathWorks). These codes will be made available in response to reasonable requests sent to the corresponding author.

#### LFP recording

LFPs were measured as the voltage difference between the two tips of a bipolar electrode (the difference in the length was 0.7 mm) and continuously recorded during daily conditioning. LFPs were amplified by 1000 times, filtered between 1 and 400 Hz, and digitized at 2034 Hz with the same RZ-5 recording system used for the recording of EMG activity.

#### Preprocessing

To remove trials with movement artifact, a threshold was defined as the across-trial mean plus 4 SD of the raw LFP signals during a 500 ms period before CS onset. This threshold was selected based on visual inspection of several datasets. If LFP signals during a 500 ms period before CS onset exceeded this threshold in a trial, the trial was excluded from further analyses. All remaining trials, regardless of the presence of CR or not, were included in the analysis, and the results were pooled. Subsequently, the power spectrum density was calculated to ensure that its shape was consistent with those reported in previous studies (Paz et al., 2008; Insel et al., 2012; Volle et al., 2016).

#### Spectrogram

Time-frequency analyses were conducted on raw LFP data during the seven differential learning sessions by means of the Morlet wavelets method, using the *ft_freqanalysis* function from the FieldTrip toolbox (Oostenveld et al., 2011). To detect changes in oscillatory activity induced by CS presentation, the power in each frequency band was divided by the averaged power during a 1 s window before the CS presentation.

#### Amplitude of CS-evoked oscillatory activity

To detect CS-induced changes in oscillatory activity, we computed instantaneous amplitude by using the Hilbert transform. This method was chosen over other methods because of its high temporal resolution. The amplitude of three predetermined frequency bands (theta, 4–12 Hz; beta, 13–30 Hz; gamma, 30–100 Hz) during the CS and interval between the CS and US (trace interval) was computed with the following steps. First, in each trial, raw LFP signals during a 2 s window around CS onset were filtered with a bandpass filter (third-order Chebyshev type 1, forward and backward for zero phase distortion using the *filtfilt* function in MATLAB). Second, the instantaneous amplitude was calculated as the absolute value of the Hilbert transform of the filtered signal. Third, the amplitude was divided by the averaged amplitude during a 500 ms window before the CS onset (see Figs. 3, 4). Fourth, the normalized amplitude was averaged in a predetermined time window. Fifth, the values were averaged across trials and then used to calculate the DI as follows:

*DI* = abs(*AMP_CSP* − *AMP_CSM*)/(*AMP_CSP* + *AMP_CSM*), where *AMP_CSP* and *AMP_CSM* are the averaged, normalized amplitude in reinforced and unreinforced trials, respectively.

#### Correlation with CR%

In each rat, the Pearson correlation coefficient (*r*) was calculated between CR% and the normalized theta amplitude to each CS type during the seven sessions of the differential learning stage. *R* values were then averaged across rats in each group.

### Experimental design and statistical analyses

#### Cell counting

The data presented are the group’s mean ± the group’s SEM across rats. Each group contained five rats. *T* tests were used to determine statistical significance (α = 0.05). For the experiment testing the possibility of a nonspecific effect of CNO, a one-way ANOVA with group as a between-subjects factor was used with a Tukey *post hoc* test.

#### EMG analysis

For the comparison of CR%, the data presented are the group’s mean for each day of conditioning ± the group’s SEM. Mixed ANOVAs with group as a between-subjects factor and session, epoch and CS type as within-subjects factors were used to determine statistical significance. The number of sessions needed for *DI_CR* to reach the significance was compared between the groups with Wilcoxon rank sum test.

#### Electrophysiological analyses

The data were presented as the mean and SEM across rats (Figs. 3*C*, 4, 5). To test the difference in CS-evoked amplitude, we ran mixed ANOVA on the DI with sessions or learning stages as a within-subjects factor and groups as a between-subjects factor. Correlations between CS-evoked theta amplitude and CR% were compared between the two groups with *t* tests.

## Results

### Excitatory pyramidal neurons in the mPFC were selectively activated by virally transduced DREADDs

**Figure 1.**
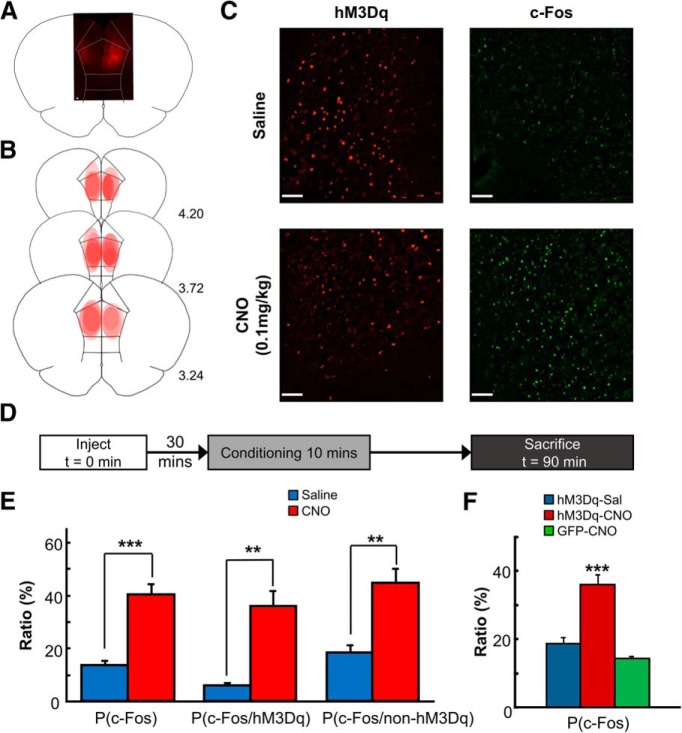
Histologic validation of chemogenetic manipulations in hM3Dq-expressing rats. ***A***, A representative photomicrograph of the mPFC of a rat with virally transduced hM3Dq-mCherry gene. hM3Dq expression (red) was observed bilaterally in the prelimbic region of mPFC. Scale bar, 100 μm. ***B***, A histologic reconstruction of the viral infection area in hM3Dq-expressing rats. Each red oval shape represents the spread of mCherry signal observed in each rat. hM3Dq was expressed in the prelimbic region with minimal spread into adjacent infralimbic and anterior cingulate cortices in all the rats included in the behavioral and neurophysiological analyses. ***C***, Representative images of hM3Dq (red) and c-Fos (green) expression in saline-treated rats (top) and CNO-treated rats (bottom). Scale bars, 100 μm. ***D***, Experimental timeline. ***E***, Cell-counting results confirmed that the proportion of c-Fos-positive cells was higher in the CNO-treated rats than in those treated with saline. The difference was significant in both hM3Dq-positive and hM3Dq-negative cells. Data are shown as mean ± SEM. **p < 0.01 ***p < 0.001 (*t* test). ***F***, The proportion of c-Fos-positive cells was significantly higher in hM3Dq-expressing rats treated with CNO (hM3Dq-CNO) than those treated with saline (hM3Dq-Sal) or GFP-expressing rats treated with CNO (GFP-CNO). Data are shown as mean ± SEM. ***p < 0.001 (one-way ANOVA).

We used AAVs to transduce the evolved human M3-muscarinic receptor (*hM3Dq*) gene into a subset of pyramidal neurons in the prelimbic region of the mPFC in adult male Long–Evans rats. The hM3Dq receptor can be activated by systemic injection of the pharmacologically inert ligand, CNO (Alexander et al., 2009). To target expression to excitatory pyramidal neurons, the *hM3Dq* gene was expressed under the control of the *CaMKIIα* promoter. The expression of hM3Dq receptors (detected by coexpression of mCherry) was restricted to the prelimbic region with very minor spread into adjacent rostral anterior cingulate and infralimbic regions (Fig. 1*A*,*B*). Among 24 rats that received the viral vector with the *hM3Dq* gene, 5 rats had insufficient spread into the prelimbic region. The data from these rats were removed from subsequent analyses.

To examine the cumulative impact of the chemogenetic manipulation and behavioral conditioning on the activity of single neurons, we monitored the expression of the immediate early gene, c-Fos, induced by subcutaneous injection of CNO or saline before the start of conditioning in 10 hM3Dq-expressing rats (Fig. 1*C*). Although many previous studies used hM3Dq to induce artificial firing (Farrell and Roth, 2013), our goal was to enhance natural responses of prefrontal neurons to incoming stimuli. Therefore, we used a low dose of CNO (0.1 mg/kg body weight), which is ∼5–30 times lower than the doses used in other studies (Garner et al., 2012; Zhan et al., 2013; Yau and McNally, 2015).

The overall number of c-Fos-expressing cells was higher in CNO-treated hM3Dq-expressing rats (*n* = 5 rats) than those treated with saline (*n* = 5; Fig. 1*E*; *t* test, *p* < 0.001). Consistent with previous reports (Garner et al., 2012; Zhan et al., 2013; Yau and McNally, 2015; Volle et al., 2016), the increase was observed regardless of hM3Dq expression: the proportion of c-Fos-expressing cells was higher in CNO-treated rats than in saline-treated rats in both hM3Dq-positive (p = 0.001) and hM3Dq-negative cells (*p* = 0.002). These results suggest that our manipulation increased the number of activated cells during the conditioning after systemic CNO injection.

Increased c-Fos expression in hM3Dq-negative cells raises a possibility that CNO may have nonspecifically increased neural activity regardless of hM3Dq expression. We confirmed that this was not the case by using a separate cohort of hM3Dq- and GFP-expressing rats that received CNO or saline injections without the conditioning (Fig. 1*F*). The proportion of c-Fos-expressing cells was significantly higher in CNO-treated hM3Dq-expressing rats (*n* = 5) compared with CNO-treated GFP-expressing rats (*n* = 5) and saline-treated hM3Dq-expressing rats (*n* = 5; one-way ANOVA, *F*_(2,12)_ = 32.150, *p* < 0.001; Tukey *post hoc* test: *p* < 0.001). The latter two groups showed a comparable proportion of c-Fos expressing cells (*p* = 0.303). These findings suggest that CNO itself was not effective in activating neurons and that the increased c-Fos expressing in hM3Dq-negative cells (Fig. 1*E*) was likely because of the excitatory synaptic connections between hM3Dq-expressing and hM3Dq-negative cells.

### Increasing prefrontal neuron activity accelerated the formation of temporal stimulus associations without compromising accuracy or flexibility

**Figure 2.**
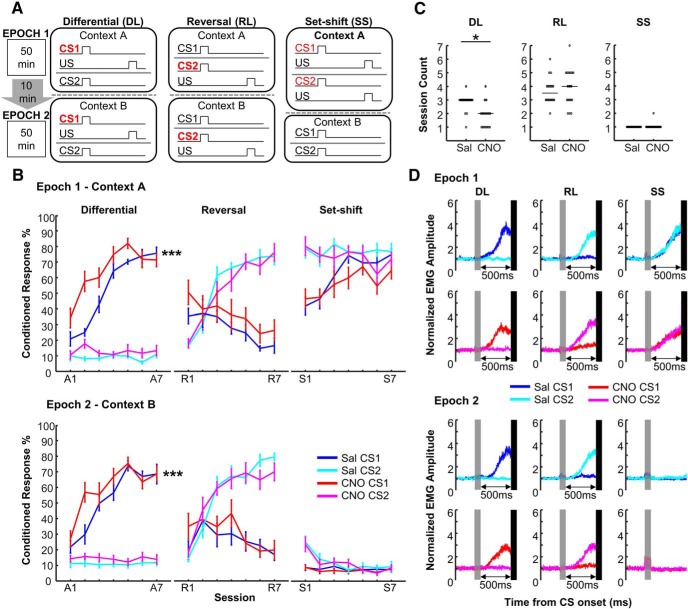
Increasing prefrontal neuron activity promoted the formation of specific temporal stimulus associations. ***A***, Schematic diagram showing stimulus contingency used during differential, reversal learning, and set-shift stages. Daily sessions consisted of two epochs during which rats received pairings of either tone or light (CS1, CS2) with eyelid shock (US) in two distinct conditioning environments (Contexts A and B). Red color shows stimuli that were predictive of the US. ***B***, In the differential learning stage (left), CNO-treated hM3Dq-expressing rats (red; *n* = 10; mean ± SEM) acquired CRs to the CS that was paired with the US (CS1+) faster than saline-treated rats (blue; *n* = 9) in both epochs. In contrast, CR expression to the other CS that was presented alone (CS2−) did not differ between saline- (cyan) and CNO-treated (magenta) rats. In the reversal stage (middle), both groups increased CR% for the newly reinforced CS2+ while concurrently decreasing CR expression for the currently non-reinforced CS1−. In the set-shift stage (right), both groups showed comparable CR% to two CS in each of two epochs while differentiating CR% to the same CS between the two epochs. ∗∗∗*p* < 0.001 (mixed ANOVA). ***C***, CNO-treated rats developed differential responding faster than saline-treated rats during differential learning, but not the other two stages. Individual data shown with the median (line). *p < 0.05 (Wilcoxon rank sum test). ***D***, Averaged normalized EMG amplitude revealed that the temporal pattern of EMG activity was comparable between the saline-treated (blue and cyan) and CNO-treated (red and magenta) rats in both epochs across the three learning stages. Gray bars depict CS presentation and black bars mask the artifact generated by the US. DL- Differential Learning; RL- Reversal Learning; SS- Set-shift.

We first examined the effect of chemogenetic activation of the mPFC on differential, reversal learning and set-shifting paradigms in trace eyeblink conditioning. Although CNO or the metabolic conversion of CNO to clozapine may have off-target effects (Gomez et al., 2017), our previous study confirmed that systemic injections of CNO to rats that did not express hM3Dq had no effect on the acquisition of conditioned responses (CRs) in trace eyeblink conditioning (Volle et al., 2016). We, therefore, used saline-treated hM3Dq-expressing animals as controls. Nineteen hM3Dq-expressing rats daily underwent two epochs of differential trace eyeblink conditioning in two distinct conditioning environments (Fig. 2*A*). Thirty minutes before the first conditioning epoch, 9 rats received systemic injection of saline, whereas 10 rats received injection of CNO (0.1 mg/kg). These rats received the same treatment across all sessions. In the differential learning stage, CNO-treated hM3Dq-expressing rats acquired CRs to a neutral stimulus (CS1+, tone or light) that was paired with mild electric shock near the eyelid (US) faster than saline-treated hM3Dq-expressing rats in both epochs (Fig. 2*B*; four-way mixed ANOVA, Epoch×Group×CS×Session interaction: *F*_(6102)_ = 0.686, *p* = 0.662; Group×CS×Session interaction: *F*_(6102)_ = 4.716, *p* < 0.001). Follow-up two-way mixed ANOVA revealed that the frequency of CR expression (CR%) to the CS1+ was higher in the CNO-treated than saline-treated rats (Group×Session interaction: *F*_(6102)_ = 5.617, p < 0.001). In contrast, the two groups showed comparable CR expression to the other CS presented alone across seven sessions (CS2−, light or tone; Group×Session interaction: *F*_(6102)_ = 1.063, *p* = 0.390; Group: *F*_(1,17)_ = 3.445, p = 0.081; Session: *F*_(6102)_ = 0.631, *p* = 0.705).

Subsequently, the rats underwent the reversal learning stage, during which the stimulus contingency of the two CS with the US was reversed in both environments. CR% to either the currently non-reinforced CS1− or the newly reinforced CS2+ did not differ between the two groups in either of the two epochs (Fig. 2*B*; Epoch×Group×CS×Session interaction: *F*_(6102)_ = 0.583, p = 0.743; Group×CS×Session interaction: *F*_(6102)_ = 1.866, *p* = 0.094). Both groups increased CR% for the CS2+ (CS×Session interaction: *F*_(6102)_ = 95.721, *p* < 0.001; follow-up one-way repeated-measure ANOVA: *F*_(6108)_ = 91.823, *p* < 0.001) while concurrently decreasing CR expression for the CS1− (*F*_(6108)_ = 9.371, *p* < 0.001).

The last set-shift stage required the rats to revise the previously acquired associations such that the conditioning environment, rather than the modality of CS, signaled which CS was predictive of the US. One CNO-treated rat was removed because of the loss of the head implant. Both groups showed comparable CR% to the two CS in each of the two epochs (Fig. 2*B*; Epoch×Group×CS×Session interaction: *F*_(6,96)_ = 0.530, *p* = 0.785; Group×CS×Session interaction: *F*_(6,96)_ =1.226, *p* = 0.300). CR%, however, differed between the two epochs (Epoch×Session interaction: *F*_(6,96)_ = 8.154, *p* < 0.001) because both groups increased CR% to both CS in Epoch 1 during which the two CS were paired with the US (follow-up one-way repeated-measure ANOVA, a main effect of session: *F*_(6102)_ = 4.604, *p* < 0.001). In contrast, they decreased CR expression to both CS in Epoch 2 during which the two CS were presented by themselves (*F*_(6102)_ = 7.768, *p* < 0.001).

Faster differential learning in CNO-treated rats was also confirmed with another measure, which was designed to quantify the speed that rats developed reliable, differential responding to the two CS types (*DI_CR* combined with random permutation tests; see Materials and Methods). Compared with saline-treated rats, CNO-treated rats took fewer sessions to significantly differentiate CR% between the two CS during the differential learning stage (Fig. 2*C*; Wilcoxon rank sum test, *p* < 0.001). In contrast, both groups took a comparable number of sessions to adjust CR expression during the reversal learning and set-shift stages (*p* = 0.423, 0.377).

In addition, the two groups showed comparable temporal patterns of EMG activity in all three stages (Fig. 2*D*), suggesting that chemogenetic mPFC activation did not change the timing or the degree to which EMG amplitude was increased after CS onset (Volle et al., 2016).

Collectively, these findings suggest that chemogenetic mPFC activation facilitated differential learning but did not affect reversal learning or set-shifting.

### CS-evoked theta amplitude was selective for the behavioral relevance of stimuli

**Figure 3.**
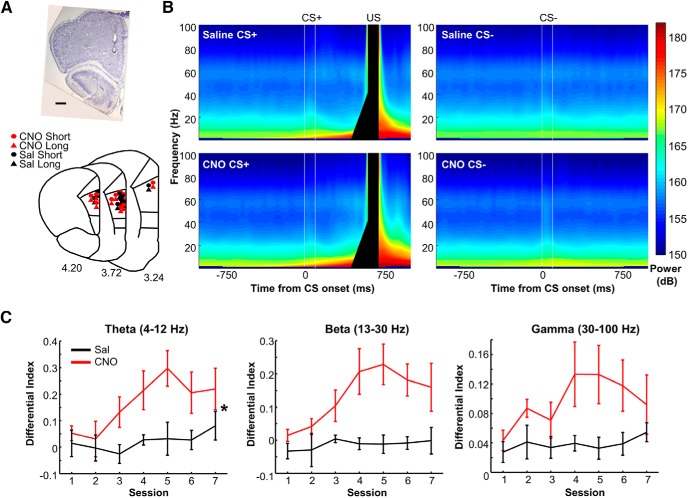
CS-evoked theta amplitude was selective for relevant temporal stimulus correlations. ***A***, Representative image of electrode tip location in the prelimbic region of mPFC (top) and histologic reconstruction of electrode locations (bottom) in the CNO-treated (red) and saline-treated (black) hM3Dq-expressing rats included in neurophysiological analyses. The locations of shorter and longer tips of bipolar electrodes are depicted as circles and triangles, respectively. Scale bar, 500 μm. ***B***, Spectrograms during the differential learning stage showing the power of oscillations averaged across sessions and rats in each group. Two white lines show CS onset and offset. Black bars mask the artifact generated by the US. ***C***, The degree of differentiation of the CS-evoked oscillatory amplitude between the reinforced and unreinforced CS during the differential learning stages (mean ± SEM; saline: *n* = 8 rats; CNO: *n* = 9 rats). The amplitude of theta band activity (4–12 Hz) differentiated two CS types, and the differentiation was stronger in CNO-treated (red) than saline-treated (black) rats. *p < 0.05 (mixed ANOVA).

To monitor changes in the network activity in responses to stimuli, we recorded LFPs from the prelimbic region of the mPFC during these conditioning sessions (Fig. 3*A*). Two rats (1 from each group) were removed because of misplaced electrodes, leaving eight saline-treated and nine CNO-treated rats for neural activity analyses. To examine whether the mPFC activity was selective for the behavioral relevance of the stimuli, we first analyzed LFPs during the differential learning stage. The CS evoked a lasting field potential response in the mPFC of both saline- and CNO-treated rats with prominent components in the theta frequency band (4–12 Hz; Fig. 3*B*). Both groups showed larger evoked responses to the CS paired with the US (CS1+) than the CS presented alone (CS2−). Moreover, after the CS1+, the stimulus-evoked responses became stronger to the expected onset of the US. To quantify these observations, raw LFPs were bandpass filtered within a predetermined frequency band, converted to instantaneous amplitude with Hilbert transform, and normalized with baseline amplitude. We then averaged the normalized amplitude during the last 100 ms time window ending at the time point closest to the US onset without any contamination of US artifact. These amplitude data were then converted to a DI in each rat to quantify the degree to which the normalized amplitude differentiated two CS types. In both groups, the DI of CS-evoked theta band activity (4–12 Hz; Fig. 3*C*, left) became stronger across seven sessions (two-way mixed ANOVA; Group×Session interaction: *F*_(6,90)_ = 1.462, *p* = 0.200; Session: *F*_(6,90)_ = 2.991, *p* = 0.010). Also, CNO-treated rats showed larger DI than saline-treated rats (Group: *F*_(1,15)_ = 7.399, *p* = 0.016). On the other hand, there was a trend that the DI of CS-evoked beta band activity (13–30 Hz) was increased in CNO-, but not saline-treated rats (Group×Session interaction: *F*_(6,90)_ = 2.184, *p* = 0.052). The DI of CS-evoked gamma band activity (30–100 Hz) did not significantly change across sessions (Group×Session interaction: *F*_(6,90)_ = 1.432, *p* = 0.211; Session: *F*_(6,90)_ = 1.840, *p* = 0.100). However, there was a trend that the DI in CNO-treated rats was larger than saline-treated rats (Group, *F*_(1,15)_ = 4.507, *p* = 0.051). These results suggest that with differential learning, CS-evoked theta amplitude became more selective for behavioral relevance of the CS and that chemogenetic activation of excitatory neurons facilitated the development of this selectivity. Based on these observations, we concentrated further analyses on theta band activity.

### Increasing prefrontal neuron activity promoted the development of ramping theta responses selective for behaviorally relevant stimuli

**Figure 4.**
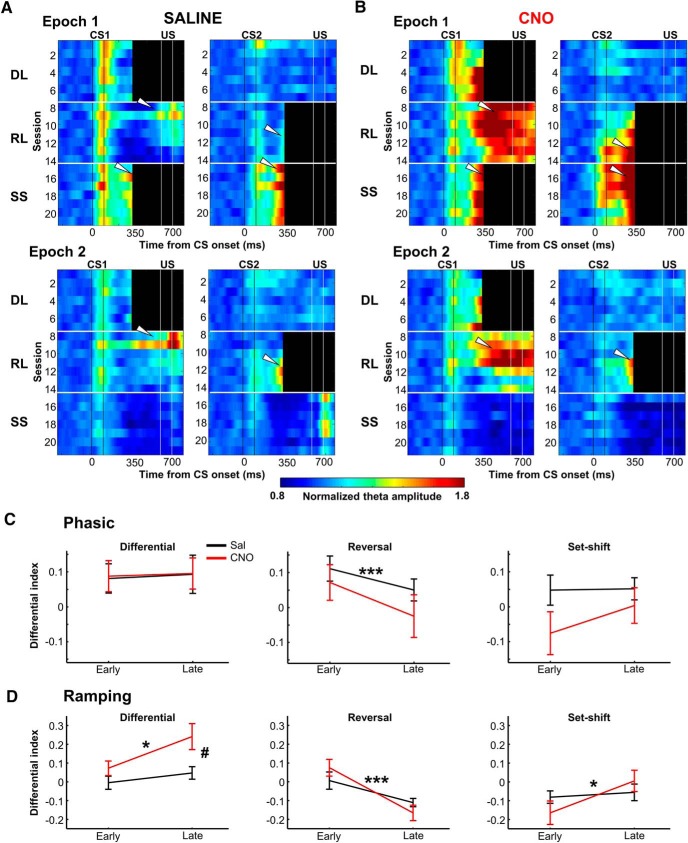
Increasing the mPFC activity promoted the development of ramping theta responses selective for behaviorally relevant stimuli. ***A***, Normalized theta amplitude to the originally reinforced CS (CS+; left) and unreinforced CS (CS−; right) in saline-treated rats is plotted over time within trials (*x*-axis) across days (*y*-axis, descending from top to bottom) in Epochs 1 (top) and 2 (bottom). Horizontal white lines separate the three stages of learning. Two vertical black and white lines show the timing of the CS and US, respectively. Black bars mask the artifact generated by the US. During the differential learning stages, theta amplitude was transiently increased during the CS+ but not the CS−. Although this phasic response did not track the subsequent change in stimulus contingency, theta amplitude came to ramp up toward the expected onset of the US during the reversal learning and set-shift stages (white arrows). ***B***, The same as A for CNO-treated rats. Within a few sessions in the differential learning stages, theta amplitude came to ramp up toward the expected US onset, and the ramping responses tracked changing stimulus contingency during the reversal learning and set-shift stages (white arrows). ***C***, The Differential Index of the phasic responses during the first three (early phase) and latter three (late phase) sessions of each learning stage (mean ± SEM; saline: *n* = 8 rats; CNO: *n* = 9 rats). ***p < 0.001 (main effect of phase in mixed ANOVA). ***D***, the same as ***C*** for the ramping responses. During differential learning, both groups improved the differentiation of the ramping responses across two phases, and the differentiation was stronger in CNO-treated rats than saline-treated rats. *p < 0.05, ***p < 0.001 (main effect of stage); #*p* < 0.05 (main effect of group). DL- Differential Learning; RL- Reversal Learning; SS- Set-shift.

Having established that stimulus-evoked theta band activity differentiated the reinforced CS from the unreinforced CS, we then examined the time course over which the selective activity was developed and later adjusted according to changing stimulus contingencies. During the first few sessions of the differential learning stage, saline-treated rats showed a stronger phasic increase after the presentation of CS1+ compared to CS2− (Fig. *4A*). Similar patterns were also observed in Epoch 2. The strong phasic response to the CS1+ was maintained during the rest of the differential learning stage. During the reversal learning stage, the phasic response continued to be stronger for the CS1− than the CS2+ despite the reversed stimulus contingency. In addition, during the first few sessions, in which the rats still frequently showed CRs to the CS1− (Fig. *2B*), theta amplitude ramped up starting at ∼100 ms before the timing at which the US was presented during the previous stage (Fig. *4A*, white arrows). This ramping response to the CS1− disappeared toward the end of the reversal stage while similar ramping responses gradually developed after the CS2+ (Fig. *4A*, last 3 sessions). During the set-shift stage, in Epoch 1 the ramping responses were observed for both CS with comparable strength though the phasic response was still stronger for the CS1+ than CS2+. The ramping response was absent in Epoch 2 in which neither of two CS was paired with the US.

In parallel to the enhanced differential learning (Fig. 2*B*,*C*), chemogenetic mPFC activation accelerated the development of the selective ramping response. From the beginning of the differential learning stage, CNO-treated rats showed strong phasic responses as well as signs of ramping responses differentiating the two CS (Fig. 4*B*). Toward the end of the differential learning stage, the differentiation of the ramping responses became stronger. During the reversal stage, the ramping response gradually became smaller for the CS1− while concomitantly becoming larger for the CS2+. During the set-shift stage, the ramping responses did not differ between the two CS types in either of the epochs. These results suggest that in both saline- and CNO-treated rats, the CS-evoked theta band activity consisted of two components: the first component sharply increased after CS onset and differentiated the CS+ from the CS− during differential learning, but not during subsequent reversal learning or set-shifting (phasic responses). The second component gradually ramped up toward the expected US onset during differential learning and tracked changing stimulus contingency during reversal learning and set-shifting (ramping responses).

To quantify these observations, we separately calculated the DI for the phasic (50–150 ms after CS onset) and ramping (250–350 ms after CS onset) responses and averaged these values across the first (early) and last (late) three sessions of each learning stage (Fig. 4*C*,*D*). During the differential learning stage, the DI of the phasic responses was comparable between the early and late phases and between the two groups (Fig. 4*C*, left; two-way mixed ANOVA, Group×Phase interaction: *F*_(1,15)_ = 0.011, *p* = 0.919; Phase: *F*_(1,15)_ = 0.237, *p* = 0.633; Group: *F*_(1,15)_ = 0.005, *p* = 0.945). During the reversal learning stage, the DI became smaller in both groups (middle; Group×Phase interaction: *F*_(1,15)_ = 2.726, *p* = 0.120; Phase: *F*_(1,15)_ = 54.474, *p* < 0.001; Group: *F*_(1,15)_ = 0.736, *p* = 0.404). During the set-shift stage, the DI was comparable between the two phases and between the two groups (right; Group×Phase interaction: *F*_(1,14)_ = 3.003, *p* = 0.105; Phase: *F*_(1,14)_ = 3.614, *p* = 0.078; Group: *F*_(1,14)_ = 1.442, *p* = 0.250).

The greater effect of chemogenetic mPFC activation was found in the ramping responses. During the differential learning stage, the DI of ramping responses was increased across the phases in both groups (Fig. 4*D*, left; Group×Phase interaction: *F*_(1,15)_ = 1.924, *p* = 0.186; Phase: *F*_(1,15)_ = 6.791, *p* = 0.020), and it was significantly larger in CNO-treated rats than saline-treated rats (Group: *F*_(1,15)_ = 6.669, *p* = 0.021). During the reversal stage, in both groups, the sign of DIs changed from positive to negative, reflecting the strengthening of the ramping responses to the CS2, which was originally non-reinforced and became newly reinforced (middle; Group×Phase interaction: *F*_(1,15)_ = 4.436, *p* = 0.052; Phase: *F*_(1,15)_ = 37.156, *p* < 0.001; Group: *F*_(1,15)_ = 0.019, *p* = 0.892). During the set-shift stage, DIs in both groups came close to zero, suggesting comparable responses to two CS types (right; Group× Phase interaction: *F*_(1,14)_ = 3.596, *p* = 0.079; Phase: *F*_(1,14)_ = 6.908, *p* = 0.020; Group: *F*_(1,14)_ = 0.400, *p* = 0.537). We also confirmed similar patterns of the group-differences in the phasic and ramping responses in Epoch 2 (data not shown). These results suggest that chemogenetic mPFC activation enhanced the differentiation of the ramping responses between the reinforced and unreinforced CS only during differential learning.

### Increasing prefrontal neuron activity strengthened correlations between the ramping activity and conditioned responses

**Figure 5.**
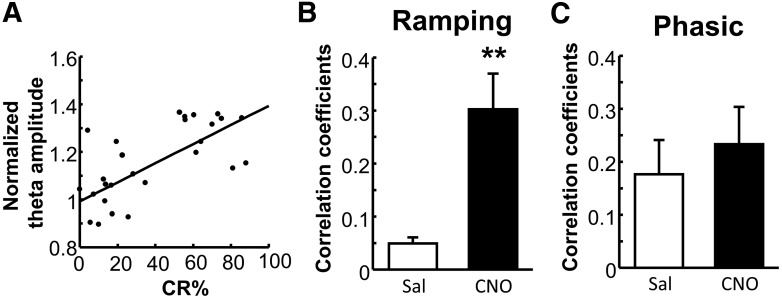
Increasing the mPFC activity strengthened the correlation between the ramping response and the frequency of conditioned responses. ***A***, Correlations of CR% with the ramping response in a representative CNO-treated rat. ***B***, Correlation coefficients between CR% and the ramping response (mean ± SEM) in CNO group (*n* = 9 rats) were significantly stronger than saline group (*n* = 8 rats). **p < 0.01 (*t* test). ***C***, The same for the phasic responses, showing the lack of the group difference.

The previous section showed that chemogenetic mPFC activation augmented the ramping activity selective for the behaviorally relevant stimuli (i.e., the CS paired with the US). To test whether the stronger ramping response was related to the faster differential learning (Fig. 2*B*), we calculated correlations between the ramping response and CR% across sessions during the differential learning stage. The majority of CNO-treated rats showed a significant correlation between the ramping response and CR% (an example rat is shown in Fig. 5*A*; for correlation coefficient in each rat, see Table 1). As a group, CNO-treated rats showed stronger correlations than saline-treated rats (Fig. 5*B*; *t* test, *p* = 0.003). In contrast, the strength of correlation between the phasic response and CR% was comparable between two groups (Fig. 5*C*; *p* = 0.560). These results suggest that the ramping, but not the phasic, theta activity was relevant for learning the behavioral relevance of stimuli and that the improved ramping activity was tightly linked to the improved differential learning.

**Table 1.** Correlation coefficient between ramping theta activity and CR% in individual rats

	Differential learning		
Rat	*R* ^2^	*F*	*P*
SAL1	0.016	0.433	0.516
SAL2	0.079	2.244	0.146
SAL3	0.040	1.089	0.306
SAL4	0.000	0.001	0.972
SAL5	0.089	2.547	0.123
SAL6	0.025	0.656	0.425
SAL7	0.065	1.796	0.192
SAL8	0.077	2.179	0.152
CNO1	0.402	17.457	<0.001
CNO2	0.037	0.990	0.329
CNO3	0.219	7.290	0.012
CNO4	0.642	46.541	<0.001
CNO5	0.246	8.477	0.007
CNO6	0.532	29.600	<0.001
CNO7	0.046	1.264	0.271
CNO8	0.305	11.398	0.002
CNO9	0.293	10.799	0.003

Correlation coefficients (*R*2), and *F* and *P* values for individual rats.

## Discussion

Although we experience an ongoing stream of events every day, the brain does not form lasting memory traces for every sequence of events that we have encountered. Seminal imaging studies in humans (Brewer et al., 1998; Wagner et al., 1998) and recent behavioral studies in rodents (Tse et al., 2011; Bero et al., 2014) implicated that the mPFC may control the strength of encoding of hippocampus-dependent memories. However, the real-time dynamics in the mPFC network that promote the encoding of relevant events remained unknown. Here, we show that the mPFC network response differentiated behaviorally relevant events from irrelevant ones, and that artificial enhancement of this exact network process facilitated the initial encoding of those events. Notably, the same manipulation did not affect the subsequent revision of these associations in the face of the changing stimulus contingency. These findings identify the mPFC network activity selective for behavioral relevance of events as a mechanism that controls the mnemonic fate of event information.

Linking two events that occur separately in time is an important aspect of everyday memory. Previous lesion and inactivation studies have shown that this ability depends on the integrity of the mPFC (Weible et al., 2003; Takehara-Nishiuchi et al., 2005; Gilmartin and Helmstetter, 2010; Gilmartin et al., 2013) and hippocampus (Solomon and Graves, 1985; Moyer et al., 1990; McEchron et al., 1998; Suh et al., 2011). In line with these early findings, we found that chemogenetic activation of the mPFC facilitated the formation of temporal stimulus associations, thereby highlighting the critical involvement of the mPFC in memory encoding dependent on the hippocampus. In contrast to the enhanced learning, chemogenetic mPFC activation did not affect rats’ ability to adjust behavioral responses to the stimuli when stimulus contingency was reversed or when the set predictive of aversive events was shifted (Fig. 2*B*). The lack of the effect on reversal and set-shifting is not because of tolerance to CNO, which might occur after repeated administration over 3 weeks, because c-Fos expression was higher in CNO-treated than saline-treated rats even on the day after the last set-shifting session (Fig. 1*E*). These observations counter the possibility that the enhanced learning is because of a general enhancement of sensory, motor processing, working memory, or attention. Rather, they support our view that increasing the activity of mPFC improves the functionality of the system specifically for the encoding of new event information.

The lack of effect on reversal and set-shifting appears to contradict with the established role of the mPFC in behavioral flexibility (Dalley et al., 2004; Floresco et al., 2009; Bissonette et al., 2013). In particular, manipulations that activate the mPFC network, such as reduced local GABA signaling via pharmacological blockade of GABA_A_ receptors (Enomoto et al., 2011) or disruption of GABAergic neurons by genetic manipulation (Cho et al., 2015), impair set-shifting, but not differential or reversal learning. Our study differed from those studies in several points, including behavioral paradigms (classical vs instrumental conditioning), valence of reinforcement (aversive event vs reward), and types of the set predictive of reinforcement (environmental context vs unimodal stimuli). In addition to these procedural differences, another critical difference is the impact of the manipulations on the balance between excitation and inhibition in local circuits. The primary consequence of our chemogenetic manipulation was the elevated activity of CaMKIIα-positive, excitatory neurons that expressed hM3Dq; however, the elevated activity was also observed in cells without hM3Dq expression (Fig. 1*E*). Considering that excitatory neurons form synaptic input onto excitatory as well as inhibitory neurons (Povysheva et al., 2008; Otsuka and Kawaguchi, 2009), the latter group of neurons likely include both excitatory and inhibitory neurons. It is, therefore, possible that the concomitant increase of glutamatergic and GABAergic signaling may lead to an optimum circuit performance for fast, accurate and flexible memory encoding. In contrast, pharmacological blockade of GABA receptors or genetic disruption of GABAergic cells reduces GABA signaling. This may be detrimental to the proper functioning of the circuit, leading to a condition similar to the dysregulation of prefrontal activity in schizophrenia and bipolar disorder (Lewis et al., 2005; Green, 2006; Lisman, 2012; Tse et al., 2015).

In parallel to enhanced differential learning, chemogenetic mPFC activation promoted the development of the ramping increase of theta amplitude toward the expected onset of the aversive stimulus. Importantly, in rats with chemogenetic mPFC activation, the ramping activity developed hand-in-hand with differential learning (Figs. 2*B*, 4*B*). Moreover, the ramping activity was correlated with the frequency of conditioned responses to the reinforced and unreinforced stimuli during differential learning (Fig. 5*A*,*B*). These observations suggest a tight link between the ramping activity and the encoding of temporal stimulus relationships. One may argue that the ramping theta activity is related to eyelid muscle contraction, which rats showed after the reinforced, but not unreinforced, stimuli. Several observations counter this argument. First, theta amplitude began to ramp up ∼250 ms after CS onset (Fig. 4), although the major increase in EMG activity occurred at ∼350 ms after CS onset (Fig. 2*D*). Second, saline-treated rats did not display ramping activity in the same temporal pattern as CNO-treated rats during the last three sessions of the differential learning stage although the two groups of rats showed comparable patterns of eyelid movement (Figs. 2*D*, 4). These findings are consistent with our view that ramping theta activity is a process that leads to associative learning rather than a consequence of eyelid movement (for additional discussion, see Volle et al., 2016).

Caution is required for the interpretation of neural activity patterns induced by gain-of-function approaches because they may be artificial and never occur in intact brains. In the present study, chemogenetic mPFC activation elevated the oscillatory activity specifically for the reinforced CS (Fig. 3*C*), in particular the ramping theta band response toward the expected onset of the aversive stimulus (Fig. 4*B*,*C*,*D*). Notably, the ramping response was also observed in control rats although it was not detectable during the differential learning stage because of US artifact (Fig. 4*A*). During the reversal and set-shift stages, the latency of the ramping activity became shorter, which made it possible to detect the ramping activity in control rats. These observations, along with similar findings in differential fear conditioning in mice (Likhtik et al., 2014) and humans (Chien et al., 2017), suggest that the mPFC develops selective theta responses to stimuli that are important enough to be sent to long-term storage. And, it is this specific computation that was augmented by chemogenetic enhancement of the activity of prefrontal excitatory neurons.

In the present study, the activity level of the mPFC was artificially elevated by chemogenetic approaches, which resulted in enhanced differential learning. This raises the question as to what controls the activity level of an intact mPFC during an experience. One factor that is known to elevate mPFC activity and facilitate learning is the similarity between new information and prior knowledge of latent patterns, categories, and rules acquired through past experiences (Wang and Morris, 2010; van Kesteren et al., 2012; Preston and Eichenbaum, 2013). For example, the mPFC becomes strongly activated while new associations are incorporated into a previously learned set of similar associations in rats (Tse et al., 2011) and humans (van Kesteren et al., 2010; Bonasia et al., 2018). Moreover, when rats encounter familiar associations in a novel environment, both memory expression and prefrontal neuron firings selective for those associations immediately generalized to the new situation (Morrissey et al., 2017). The present finding expands on the previous behavioral and neurophysiological evidence by providing a proof of concept that the elevated mPFC activity induces a network state conducive to fast, accurate, and flexible memory encoding. Therefore, one role of the mPFC would be to continuously monitor the relevance of events based on their similarity to existing knowledge structure and then send the relevancy signal to promote the encoding of those events in the hippocampus. This top-down modulation of memory encoding contrasts with the bottom-up modulation based on extrinsic factors, such as novelty and emotional arousal of experiences, which depends on the locus coeruleus (Aston-Jones and Bloom, 1981; Sara et al., 1994; Takeuchi et al., 2016; Wagatsuma et al., 2018) and amygdala (McGaugh et al., 1996, 2002; McIntyre et al., 2005).

In summary, we found that enhanced mPFC activity facilitates the accurate and flexible learning of stimulus associations by promoting the development of mPFC network activity selective for behavioral relevance of stimuli. Future studies need to identify the long-range pathways through which prefrontal relevancy signals control memory encoding processes in the hippocampus. Although monosynaptic projections from the mPFC to the hippocampus are limited (Jones and Witter, 2007; but see Rajasethupathy et al., 2015 in mice), several mPFC efferent regions, such as the entorhinal cortex (Ryou et al., 2001; Brun et al., 2008; Lu et al., 2013) and nucleus reuniens (Ito et al., 2015; Hallock et al., 2016), are capable of modifying firing patterns of hippocampal neurons. From a clinical perspective, the present finding identifies manipulations of the mPFC’s activity level as a way to tap into an intrinsic memory encoding mechanism. This raises the potential of activating the mPFC as a novel strategy for cognitive enhancement to treat the growing elderly population facing memory disabilities.

## Synthesis

Reviewing Editor: Mark Baxter, Mount Sinai School of Medicine Decisions are customarily a result of the Reviewing Editor and the peer reviewers coming together and discussing their recommendations until a consensus is reached. When revisions are invited, a fact-based synthesis statement explaining their decision and outlining what is needed to prepare a revision will be listed below. The following reviewer(s) agreed to reveal their identity: NONE. Note: If this manuscript was transferred from JNeurosci and a decision was made to accept the manuscript without peer review, a brief statement to this effect will instead be what is listed below.

I appreciate the thorough response of the authors to the comments from the previous reviewers. I have one concern related to the data analyses and treatment of samples. Multiple measures from individual rats are not independent from one another - for example, multiple images of c-fos staining from the same brain - and cannot be treated as independent observations in an ANOVA model. This violates independence assumptions of ANOVA and inflates type I error rates. These kinds of data are more appropriately addressed with statistical models for nested data (Aarts et al., 2014, Nature Neuroscience), for instance linear mixed models. In the case of the c-fos data, if I understand the manuscript correctly, there are 2 rats each in short/long delay x CNO/saline cells of a 2 x 2 design; sections analyzed and/or images taken are nested within rats, and the appropriate error term for comparison should be based on the number of rats and not the number of sections/images. The same approach should be applied to the analyses of neurophysiological measures (CS-evoked theta amplitude and ramping response): multiple sessions from individual rats (lines 510-514) are not independent from one another and treating them that way in an ANOVA model is inappropriate.

In general, the manuscript should make it more clear how many rats are in each condition for each experiment and manipulation. The subjects section specifies 24 rats, of which 5 had insufficient virus injections (lines 399-400). This appears to correspond with the drug assignments for conditioning (line 235) which specifies a total of 19 rats. 2 additional (?) rats had misplaced electrodes (lines 196-197) and were excluded from analysis; this appears to be consistent with the 8 saline- and 9-CNO treated rats cited on line 511. I think it would be clearer to state in the subjects section that of 24 rats, 5 had insufficient virus injections and 2 had misplaced electrodes, so all data presented are from 17 rats (8 saline and 9 CNO) and using this consistently throughout the paper. If some of the rats with misplaced electrodes have data shown in the c-Fos experiment, this can be indicated here as well.

minor points:

- lines 129-130 specify a five week period between virus injection and electrode surgeries and line 143 specifies six weeks; whichever one of these is inaccurate should be corrected

- for the group effect F(1,17) = 155.178, p = 0.081 on line 462 either the F statistic or the p-value is in error

- the figure 1B caption should make clear what is being shown - is the intensity of red color related to density of mCherry staining or the number of rats that have mCherry-positive neurons in a particular region?

## Author Response

We are excited to offer our manuscript, titled “Prefrontal theta oscillations promote selective encoding of relevant events,” which we would like to be considered for publication in eNeuro. This manuscript has been reviewed in Journal of Neuroscience and recommended for transfer to eNeuro. We have followed the instruction that you kindly sent to us on August 14th. Please find our responses to the reviewers' comments detailed below. Changes that were made in the manuscript are highlighted in red. I believe that we have successfully addressed the concerns raised and look forward to hearing of your decision.

Sincerely,

Kaori Takehara-Nishiuchi

Associate professor

Department of Psychology

University of Toronto

Reviewer #1:

1) In the DREADDs neuronal activation section measuring c-Fos, it is unclear how many rats were in each group. The text states 8, however, how were 8 rats divided by 4 conditions (sal-short, sal-long, CNo-short, CNO-long)? Also, the statistical analysis reports a repeated measure, which I suspect refers to the hM3Dq and non-hM3Dq expressing cells, however this should be made much clearer in the text. What is P(c-Fos)? More detail and clarity is needed here. This section ends with “our manipulation increased the number of activated neurons during the conditioning....' But one is left wondering if the data show a non-selective effect of CNO. How would this affect other areas??

[Reply] We appreciate the reviewer's comment and have clarified this point. Eight DREADD-expressing rats were used for our primary c-Fos cell counting data, where four rats received saline injections and the other four CNO injections. Of these rats, half were exposed to the short-wait and the other half were exposed to the long-wait. Four images were taken per rat, and two-way ANOVAs were performed across images, where treatment group and wait-duration were between-subjects factors. Stating that we performed a repeated measure ANOVA was an error, and we have updated the corresponding sections in the text (Page 16, Lines 2-3; Page 18, Line 12). P(c-Fos) refers to the proportion of cells expressing c-Fos: It was calculated as the number of c-Fos expressing cells that were also DAPI-positive divided by the total number of DAPI-positive cells. We have included this in the Methods section (Page 10, Lines 14-16).

As for the increased c-Fos expression in cells without hM3Dq expression, our interpretation is that these cells receive excitatory inputs from hM3Dq-expressing cells that were strongly activated during the conditioning. However, the reviewer raised an important alternative that CNO activated neurons regardless of hM3Dq expression. To address this point, we conducted an additional histological analysis with a separate cohort of rats. Six transgene expressing rats were randomly assigned to one of three groups: hM3Dq-Sal, hM3Dq-CNO, GFP-CNO. Animals were injected with either CNO or saline and perfused 90 minutes after. Unlike our original experiment, these rats stayed in their homecage after CNO injection (i.e., did not receive the conditioning). Our results show that hM3Dq-CNO rats had significantly higher P(c-Fos) than hM3Dq-Sal or GFP-CNO rats (one-way ANOVA (F(2,21) = 28.373 p &lt; 0.001, Tukey post hoc test: ps &lt; 0.001). The latter two groups did not differ in the proportion of c-Fos expressing cells (p = 0.377). This result suggests that CNO alone is not sufficient to activate neurons in the absence of the hM3Dq receptor, supporting our view that CNO treatment increased the proportion of neurons activated during the conditioning regardless of hM3Dq expression due to excitatory connections between local neurons. To make this point clear, we added a new figure (Figure 1F) and several sentences in the Results (Page 18, Lines 24-25; Page 19, Lines 1-9).

2) Fig 3C - gamma at CS - not convincing at all as presented. I understand the desire to keep the normalized amplitude scale the same for each frequency, however, the effect looks very questionable. 

[Reply] In response to this comment we have changed the scale of the y axes in Figure 3C. Two-way Mixed ANOVA showed that CS-evoked gamma amplitude significantly differed between two CS types (CS×Group interaction, F(1,117) = 1.389, p = 0.241; CS, F(1,117) = 19.813, p &lt; 0.001) and that this differentiation was augmented with CNO treatment (Group, F(1,117) = 7.083, p = 0.009).

3) What does 'mnemonic value' mean? Can a less ambiguous term be used, e.g., expectancy, association? Also the US is delivered during these trials so there may be nothing mnemonic here.

[Reply] In the original version of the manuscript, we used ”mnemonic value“ to describe the likelihood that a certain event is converted into a stable long-term memory trace. The strength of this encoding process is known to be controlled by a number of factors, one of which is the behavioral relevance of the event. In the present study, the behavioral relevance of neutral events (i.e., CS) was manipulated by changing their associability with an aversive event. According to the reviewer's suggestion, we replaced ”mnemonic value“ to ”behavioral relevance“ throughout the manuscript.

As for the second point, we analyzed behavior and neural activity that occurred after the onset of a neutral stimulus (CS) but before the onset of an aversive event (US). Thus, any changes in the CS-evoked behavior and neural activity across conditioning sessions are driven by internal, mnemonic processes that signal the upcoming behaviorally relevant event.

4) The significance of the phasic vs. ramping theta responses need to be addressed. 

[Reply] As the reviewer pointed out, the identification of two distinct components in CS-evoked theta responses, phasic and ramping responses, is one of the main findings in this study. Several critical differences between these components were identified. Firstly, the ramping, but not phasic, responses strongly differentiated the reinforced CS from the unreinforced CS and tracked the subsequent changes in stimulus contingency. Secondly, the chemogenetic mPFC activation augmented the differentiation of these ramping responses while its effect on the phasic response was marginal. Third, the chemogenetic mPFC activation enhanced the correlations of the task performance with the ramping response but not with the phasic response. To communicate these findings better, we revised the results section (Page 24, Line 22 to Page 26, Line 24).

5) P. 23 - ” in Epoch 1, the theta response to the CS2+...“. I was left with the impression that CS2 was CS2- in epoch 1 and CS2+ in epoch 2. Perhaps this clarification.

[Reply] All three stages included both epoch 1 and epoch 2 (Figure 2A). In the first and second stages, the same stimulus contingency was used in both epochs while the contingency differed between the two epochs in the third stage. Specifically, in the first stage (differential learning) CS1+ was paired with US, and CS2- was presented alone in both epoch 1 as well as epoch 2. The reversal stage follows the same pattern where contingencies in epoch 1 and 2 are the same, with CS1- unpaired and CS2+ now paired with US. In the third stage (set-shift), CS1+ and CS2+ were paired with US in epoch 1 and CS1- and CS2- were presented alone in epoch 2. The sentence picked out by the reviewer regarding CS2+ refers to the reversal stage, as indicated in the lead-in to this sentence. We have revised Figure 2A to make this more clear.

6) The results section on the pattern of ramping activity for the stimulation group is rather confusing and needs to be streamlined. There are no differences between the CSs for the CNO rats, but differences at CS1+ between sal and CNO groups, and no differences at CS2-, and there is the emergence of differences between the CSs later. 

[Reply] According to the reviewer's suggestion, we split off the paragraph into two, one describing the difference in theta amplitude between CS+ and CS- in CNO group (Page 24, Lines 10-25; Page 25, Lines 1-3) and the other describing the difference between CNO and Saline groups (Page 25, Lines 5-25; Page 26, Lines 1-11). For the latter, we added a new figure (Figure 6). We appreciate this suggestion because it significantly improved the clarity of the result section.

7) The statistics are reported at differential temporal epochs (e.g., 0-359ms, 86-180ms, 1130-350ms etc), which makes it confusing and hard to find coherence in the data. Are these different temporal periods meaningful or just where differences are found?

[Reply] This analytical approach was instrumental in identifying the phasic and ramping components of theta band activity. Typically, neural activity analyses divide time into discrete bins of a set duration. Averaging the amplitude in preassigned bins produces an amplitude estimate that depends not only on the size of the time bins, but also on their placement. To avoid this arbitrariness, we ran a permutation test at each time point to detect the difference in amplitude of theta band activity between CS+ and CS-. This approach enabled us to detect exactly when theta amplitude becomes selective for behavioral relevance of stimuli, without any a priori assumption. As a result, we were able to identify two distinct components which were differentially affected by stimulus contingency and the chemogenetic manipulation. To make this point clear, we added several sentences to Methods (Page 16, Lines 20-24) and Result section (Page 23, Lines 15-17).

8) Neural activity to behaviour correlations are very unfocussed and need a lead-in. Why should phasic and ramping activity exhibit correlations with different parts of the task? Why should we care about the other tasks (reversals and set-shifts) if PL stimulation did nothing in those tasks? Are these correlations meaningful? 

[Reply] The goal of the analysis was to demonstrate that enhanced ramping responses were related to the faster differential learning in CNO-treated rats. According to the reviewer's suggestion, we removed the correlation data in the reversal and set-shift stages (Figure 7) and rewrote the section to communicate the motivation behind this analysis better (Page 24, Line 22 to Page 26, Line 24).

9) Discussion: as there were no effects on reversals and set-shift, it is hard justify that the present results 'facilitate the accurate, flexible encoring of ..events'. 

[Reply] During the differential learning stage, animals need to form a new association while they need to revise these previously formed associations during the subsequent reversal learning and set-shifting. Our argument is that the enhanced mPFC activity selectively facilitated the initial encoding of the new association without concomitantly increasing erroneous formation of irrelevant associations or being insensitive to subsequent changes in event contingency. To make this point clear, we rephrased the sentence (Page 27, Line 13).

10) Based on the details provided in the theta amplitude portion of the results, I am unable to understand how the degrees of freedom are F(1,124). If there are 17 rats in total with a between-subjects factor of treatment (2 levels) and a within-subjects factor of sessions (7 levels), the degrees of freedom should be F(6, 90) with the df of the interaction term: 6 = (2-1)(7-1) and the residual df: 90 = (17-2)(7-1). Some clarification is needed here. 

[Reply] In each rat, we treated the data from each of seven sessions as independent samples (saline, 7 sessions × 8 rats = 56 samples; CNO, 7 sessions × 9 rats = 63 samples). We then compared these samples using two-way Mixed ANOVAs with group as a between-subjects factor and CS type as a within-subjects factor. Since there are 119 samples in total, with a between-subjects factor of treatment (2 levels) and a within-subjects factor of CS type (2 levels), the degrees of freedom would be F(1,117) with interaction df: 1 = (2-1)(2-1) and the residual df: 117 = (119-2)(2-1). We have included another sentence to clarify this point (Page 22, Lines 6-10).

Minor Issues:

1) Tissue collection was done after behavioural testing where animals were submitted to a conditioning session either 30 min or 90 min after CNO or saline injection. Are the groups counterbalanced based on their treatment history? 

[Reply] Rats were counterbalanced during behavioral testing and received the same drug assignment during the entirety of the conditioning, likewise the drug assignment was maintained for tissue collection. We have included this information in the Methods section (Page 8, Lines 8-10).

2) P6 - how long was the incubation period? 

[Reply] It was a minimum of five weeks. This information has been included in the Methods section (Page 6, Line 22).

Reviewer #2:

1) Introduction: The functions of the prelimbic cortex in encoding outcome expectancies or stimulus-outcome associations, as described in the introduction (second paragraph), sound a lot like some descriptions of the orbitofrontal cortex. See Niv, Shoenbaum, Gremel, etc. How is the prelimbic cortex unique? I think this information should be made very clear early in the manuscript. 

[Reply] We agree that our working hypothesis on the function of the prelimbic cortex shares some commonality to the function of the orbitofrontal cortex (OFC) proposed by others (Wilson et al., 2014; Wikenheiser and Schoenbaum, 2016). In fact, one paper reports the transcriptional changes in the OFC at the time of encoding in social transmission of food preference task (Lesburgueres et al., 2011), suggesting that the two regions might be capable of fulfilling the same role during memory encoding. Also, in a simple task context, the selectivity of neuron firings is very similar between the two regions (Schoenbaum and Eichenbaum, 1995; Baeg et al., 2001; Pratt and Mizumori, 2001; Gilmartin and McEchron, 2005; Hosokawa et al., 2007; Takehara-Nishiuchi and McNaughton, 2008; Morrison and Salzman, 2009; Insel and Barnes, 2015). Typically, the neural selectivity in the mPFC is interpreted as representations of learned mappings between environmental stimuli and adaptive behavior, i.e., rules (Euston et al., 2012; Eichenbaum, 2017). Wilson et al. (2014) posit that the OFC does not represent rules itself but constructs the current state with the set of rules that are most relevant to the current position within a task. This idea is supported by recent detailed characterizations of neuron firings in OFC during a task that included learning of multiple reward contingency and reversal (Wikenheiser et al., 2017). Future investigation is needed to address whether mPFC neurons lack the selectivity for the task state. Nonetheless, we are not well positioned to discuss potential functional differences between the mPFC and OFC in detail because we did not manipulate the OFC and because no study has examined the involvement of the OFC in trace eyeblink conditioning. We, however, expanded the introduction by incorporating several empirical and theoretical papers on the OFC (Page 4, Lines 13-16 and 22-24; Page 5, Lines 1-10).

2) Also in the introductions, the authors might consider a very brief phrase indicating what theta band activity is thought to reflect, for readers who are not familiar. It may also be helpful to some readers if ”mnemonic value“ was defined. 

[Reply] We appreciate these comments. We used theta band activity as a measure of the changes in the network activity in response to stimuli. To make this point clear, we added a new sentence in the introduction (Page 5, Lines 17-18).

We originally used “mnemonic value” to describe the likelihood that a certain event is converted into a stable long-term memory trace. Because the other reviewer also found this term to be ambiguous, we replaced “mnemonic value” with “behavioral relevance” throughout the manuscript.

3) Page 15 - “data presented is” should be “data presented are” throughout.

[Reply] We corrected the errors, thank you.

4) Page 17 - I understand the rationale for using a low dose of CNO to increase sensitivity of PL neurons to incoming stimuli, but I don't understand how the c-fos data suggest that this strategy was effective. Couldn't the elevated c-fos signal in DREADD-infected neurons reflect non-specific firing, which then stimulates adjacent uninfected neurons? Perhaps these points are meant to be unconnected?

[Reply] In the c-Fos experiment, rats were sacrificed after they received 30-minutes of trace eyeblink conditioning. Therefore, the group difference shows the effect of CNO on conditioning-induced expression of c-Fos, supporting our view that CNO enhanced conditioning-induced neural processes. To make this point clearer, we added several sentences in Methods (Page 10, Lines 16-24) and Results sections (Page 18, Lines 24-25; Page 19, Lines 1-9).

5) Also pages 16-17, I am having a hard time understanding how 19 DREADD-expressing rats translates to the degrees of freedom reported on page 17, and also, how a repeating measure was used. What's the repeated measure? The language “overall number” leads me to think that the ANOVA is comparing the four bars on the left side of 1E, which I don't think includes a repeating measure.

[Reply] We appreciate the reviewer's comment and have clarified this point. Eight DREADD-expressing rats were used for the hM3Dq cell counting data, where four rats received saline injections and the other four CNO injections. Of these rats, half were exposed to the short-wait and the other half were exposed to the long-wait. Four images were taken per rat, and two-way ANOVAs were performed across images, where treatment group and wait-duration were between-subjects factors. In the original version of the manuscript, we erroneously stated that a repeated measure was used. We have corrected the error (Page 18, Line 12).

## References

[B1] Alexander GM, Rogan SC, Abbas AI, Armbruster BN, Pei Y, Allen JA, Nonneman RJ, Hartmann J, Moy SS, Nicolelis MA, McNamara JO, Roth BL (2009) Remote control of neuronal activity in transgenic mice expressing evolved G protein-coupled receptors. Neuron 63:27–39. 10.1016/j.neuron.2009.06.014 19607790 PMC2751885

[B2] Armbruster BN, Li X, Pausch MH, Herlitze S, Roth BL (2007) Evolving the lock to fit the key to create a family of G protein-coupled receptors potently activated by an inert ligand. Proc Natl Acad Sci U S A 104:5163–5168. 10.1073/pnas.0700293104 17360345 PMC1829280

[B3] Aston-Jones G, Bloom FE (1981) Norepinephrine-containing locus coeruleus neurons in behaving rats exhibit pronounced responses to non-noxious environmental stimuli. J Neurosci 1:887–900. 10.1523/JNEUROSCI.01-08-00887.1981 7346593 PMC6564231

[B4] Baeg EH, Kim YB, Jang J, Kim HT, Mook-Jung I, Jung MW (2001) Fast spiking and regular spiking neural correlates of fear conditioning in the medial prefrontal cortex of the rat. Cereb Cortex 11:441–451. 10.1093/cercor/11.5.441 11313296

[B5] Bero AW, Meng J, Cho S, Shen AH, Canter RG, Ericsson M, Tsai LH (2014) Early remodeling of the neocortex upon episodic memory encoding. Proc Natl Acad Sci U S A 111:11852–11857. 10.1073/pnas.1408378111 25071187 PMC4136596

[B6] Bissonette GB, Powell EM, Roesch MR (2013) Neural structures underlying set-shifting: roles of medial prefrontal cortex and anterior cingulate cortex. Behav Brain Res 250:91–101. 10.1016/j.bbr.2013.04.037 23664821 PMC3708542

[B7] Bonasia K, Sekeres MJ, Gilboa A, Grady CL, Winocur G, Moscovitch M (2018) Prior knowledge modulates the neural substrates of encoding and retrieving naturalistic events at short and long delays. Neurobiol Learn Mem 153:26-39. 10.1016/j.nlm.2018.02.017 29474955

[B8] Brewer JB, Zhao Z, Desmond JE, Glover GH, Gabrieli JD (1998) Making memories: brain activity that predicts how well visual experience will be remembered. Science 281:1185–1187. 10.1126/science.281.5380.1185 9712581

[B9] Brun VH, Leutgeb S, Wu H-Q, Schwarcz R, Witter MP, Moser EI, Moser MB (2008) Impaired spatial representation in CA1 after lesion of direct input from entorhinal cortex. Neuron 57:290–302. 10.1016/j.neuron.2007.11.034 18215625

[B10] Chien JH, Colloca L, Korzeniewska A, Cheng JJ, Campbell CM, Hillis AE, Lenz FA (2017) Oscillatory EEG activity induced by conditioning stimuli during fear conditioning reflects salience and valence of these stimuli more than expectancy. Neuroscience 346:81–93. 10.1016/j.neuroscience.2016.12.047 28077278 PMC5426483

[B11] Cho KKA, Hoch R, Lee AT, Patel T, Rubenstein JLR, Sohal VS (2015) Gamma rhythms link prefrontal interneuron dysfunction with cognitive inflexibility in Dlx5/6^+/−^ mice. Neuron 85:1332–1343. 10.1016/j.neuron.2015.02.019 25754826 PMC4503262

[B12] Dalley JW, Cardinal RN, Robbins TW (2004) Prefrontal executive and cognitive functions in rodents: neural and neurochemical substrates. Neurosci Biobehav Rev 28:771–784. 10.1016/j.neubiorev.2004.09.006 15555683

[B13] Eichenbaum H (2000) A cortical-hippocampal system for declarative memory. Nat Rev Neurosci 1:41–50. 10.1038/35036213 11252767

[B14] Eichenbaum H (2017) Prefrontal-hippocampal interactions in episodic memory. Nat Rev Neurosci 18:547–558. 10.1038/nrn.2017.74 28655882

[B15] Enomoto T, Tse MT, Floresco SB (2011) Reducing prefrontal gamma-aminobutyric acid activity induces cognitive, behavioral, and dopaminergic abnormalities that resemble schizophrenia. Biol Psychiatry 69:432–441. 10.1016/j.biopsych.2010.09.038 21146155

[B16] Euston DR, Gruber AJ, McNaughton BL (2012) The role of medial prefrontal cortex in memory and decision making. Neuron 76:1057–1070. 10.1016/j.neuron.2012.12.002 23259943 PMC3562704

[B17] Farrell MS, Roth BL (2013) Pharmacosynthetics: reimagining the pharmacogenetic approach. Brain Res 1511:6–20. 10.1016/j.Brainres.2012.09.043 23063887 PMC3562395

[B18] Floresco SB, Zhang Y, Enomoto T (2009) Neural circuits subserving behavioral flexibility and their relevance to schizophrenia. Behav Brain Res 204:396–409. 10.1016/j.bbr.2008.12.001 19110006

[B19] Frankland PW, Bontempi B (2005) The organization of recent and remote memories. Nat Rev Neurosci 6:119–130. 10.1038/nrn1607 15685217

[B20] Garner AR, Rowland DC, Hwang SY, Baumgaertel K, Roth BL, Kentros C, Mayford M (2012) Generation of a synthetic memory trace. Science 335:1513–1516. 10.1126/science.1214985 22442487 PMC3956300

[B21] Gilmartin MR, Helmstetter FJ (2010) Trace and contextual fear conditioning require neural activity and NMDA receptor-dependent transmission in the medial prefrontal cortex. Learn Mem 17:289–296. 10.1101/lm.1597410 20504949 PMC2884289

[B22] Gilmartin MR, McEchron MD (2005) Single neurons in the medial prefrontal cortex of the rat exhibit tonic and phasic coding during trace fear conditioning. Behav Neurosci 119:1496–1510. 10.1037/0735-7044.119.6.1496 16420154

[B23] Gilmartin MR, Miyawaki H, Helmstetter FJ, Diba K (2013) Prefrontal activity links nonoverlapping events in memory. J Neurosci 33:10910–10914. 10.1523/JNEUROSCI.0144-13.2013 23804110 PMC3693060

[B24] Gomez JL, Bonaventura J, Lesniak W, Mathews WB, Sysa-Shah P, Rodriguez LA, Ellis RJ, Richie CT, Harvey BK, Dannals RF, Pomper MG, Bonci A, Michaelides M (2017) Chemogenetics revealed: DREADD occupancy and activation via converted clozapine. Science 357:503–507. 10.1126/science.aan2475 28774929 PMC7309169

[B25] Green MF (2006) Cognitive impairment and functional outcome in schizophrenia and bipolar disorder. J Clin Psychiatry 67:3–8; discussion 36-42. 16965182

[B26] Hallock HL, Wang A, Griffin AL (2016) Ventral midline thalamus is critical for hippocampal-prefrontal synchrony and spatial working memory. J Neurosci 36:8372–8389. 10.1523/JNEUROSCI.0991-16.2016 27511010 PMC4978800

[B27] Hosokawa T, Kato K, Inoue M, Mikami A (2007) Neurons in the macaque orbitofrontal cortex code relative preference of both rewarding and aversive outcomes. Neurosci Res 57:434–445. 10.1016/j.neures.2006.12.003 17239463

[B28] Insel N, Barnes CA (2015) Differential activation of fast-spiking and regular-firing neuron populations during movement and reward in the dorsal medial frontal cortex. Cereb Cortex 25:2631–2647. 10.1093/cercor/bhu062 24700585 PMC4566002

[B29] Insel N, Patron LA, Hoang LT, Nematollahi S, Schimanski LA, Lipa P, Barnes CA (2012) Reduced gamma frequency in the medial frontal cortex of aged rats during behavior and rest: implications for age-related behavioral slowing. J Neurosci 32:16331–16344. 10.1523/JNEUROSCI.1577-12.2012 23152616 PMC3509949

[B30] Ito HT, Zhang SJ, Witter MP, Moser EI, Moser MB (2015) A prefrontal-thalamo-hippocampal circuit for goal-directed spatial navigation. Nature 522:50–55. 10.1038/nature14396 26017312

[B31] Jones BF, Witter MP (2007) Cingulate cortex projections to the parahippocampal region and hippocampal formation in the rat. Hippocampus 17:957–976. 10.1002/hipo.20330 17598159

[B32] Kao Y-C, Davis ES, Gabrieli JDE (2005) Neural correlates of actual and predicted memory formation. Nat Neurosci 8:1776–1783. 10.1038/nn1595 16286927

[B33] Kirchhoff BA, Wagner AD, Maril A, Stern CE (2000) Prefrontal-temporal circuitry for episodic encoding and subsequent memory. J Neurosci 20:6173–6180. 10.1523/JNEUROSCI.20-16-06173.2000 10934267 PMC6772579

[B84] Kitamura T, Ogawa SK, Roy DS, Okuyama T, Morrissey MD, Smith LM, Redondo RL, Tonegawa S (2017) Engrams and circuits crucial for systems consolidation of a memory. Science 356:73–78. 10.1126/science.aam6808 28386011 PMC5493329

[B34] Lesburguères E, Gobbo OL, Alaux-Cantin S, Hambucken A, Trifilieff P, Bontempi B (2011) Early tagging of cortical networks is required for the formation of enduring associative memory. Science 331:924–928. 10.1126/science.1196164 21330548

[B35] Lewis DA, Hashimoto T, Volk DW (2005) Cortical inhibitory neurons and schizophrenia. Nat Rev Neurosci 6:312–324. 10.1038/nrn1648 15803162

[B36] Likhtik E, Stujenske JM, Topiwala MA, Harris AZ, Gordon JA (2014) Prefrontal entrainment of amygdala activity signals safety in learned fear and innate anxiety. Nat Neurosci 17:106–113. 10.1038/nn.3582 24241397 PMC4035371

[B37] Lisman J (2012) Excitation, inhibition, local oscillations, or large-scale loops: what causes the symptoms of schizophrenia?. Curr Opin Neurobiol 22:537–544. 10.1016/j.conb.2011.10.018 22079494 PMC3302967

[B38] Lu L, Leutgeb JK, Tsao A, Henriksen EJ, Leutgeb S, Barnes CA, Witter MP, Moser M-B, Moser EI (2013) Impaired hippocampal rate coding after lesions of the lateral entorhinal cortex. Nat Neurosci 16:1085–1093. 10.1038/nn.3462 23852116

[B41] McEchron MD, Bouwmeester H, Tseng W, Weiss C, Disterhoft JF (1998) Hippocampectomy disrupts auditory trace fear conditioning and contextual fear conditioning in the rat. Hippocampus 8:638–646. 10.1002/(SICI)1098-1063(1998)8:6&amp;lt;638::AID-HIPO6&amp;gt;3.0.CO;2-Q 9882021

[B42] McGaugh JL, Cahill L, Roozendaal B (1996) Involvement of the amygdala in memory storage: interaction with other brain systems. Proc Natl Acad Sci U S A 93:13508–13514. 8942964 10.1073/pnas.93.24.13508 PMC33638

[B43] McGaugh JL, McIntyre CK, Power AE (2002) Amygdala modulation of memory consolidation: interaction with other brain systems. Neurobiol Learn Mem 78:539–552. 12559833 10.1006/nlme.2002.4082

[B44] McIntyre CK, Miyashita T, Setlow B, Marjon KD, Steward O, Guzowski JF, McGaugh JL (2005) Memory-influencing intra-basolateral amygdala drug infusions modulate expression of Arc protein in the hippocampus. Proc Natl Acad Sci U S A 102:10718–10723. 10.1073/pnas.0504436102 16020527 PMC1175582

[B45] Miller EK, Cohen JD (2001) An integrative theory of prefrontal cortex function. Annu Rev Neurosci 24:167–202. 10.1146/annurev.neuro.24.1.167 11283309

[B46] Morrison SE, Salzman CD (2009) The convergence of information about rewarding and aversive stimuli in single neurons. J Neurosci 29:11471–11483. 10.1523/JNEUROSCI.1815-09.2009 19759296 PMC2782596

[B47] Morrissey MD, Insel N, Takehara-Nishiuchi K (2017) Generalizable knowledge outweighs incidental details in prefrontal ensemble code over time. eLife 6:e22177. 10.7554/eLife.22177 28195037 PMC5308892

[B48] Moyer JR, Deyo RA, Disterhoft JF (1990) Hippocampectomy disrupts trace eye-blink conditioning in rabbits. Behav Neurosci 104:243–252. 2346619 10.1037//0735-7044.104.2.243

[B49] Nadel L, Moscovitch M (1997) Memory consolidation, retrograde amnesia and the hippocampal complex. Curr Opin Neurobiol 7:217–227. 9142752 10.1016/s0959-4388(97)80010-4

[B50] Oostenveld R, Fries P, Maris E, Schoffelen JM (2011) FieldTrip: open source software for advanced analysis of MEG, EEG, and invasive electrophysiological data. Comput Intell Neurosci 2011:156869. 10.1155/2011/156869 21253357 PMC3021840

[B51] Otsuka T, Kawaguchi Y (2009) Cortical inhibitory cell types differentially form intralaminar and interlaminar subnetworks with excitatory neurons. J Neurosci 29:10533–10540. 10.1523/JNEUROSCI.2219-09.2009 19710306 PMC6665698

[B52] Otten LJ, Henson RNA, Rugg MD (2002) State-related and item-related neural correlates of successful memory encoding. Nat Neurosci 5:1339–1344. 10.1038/nn967 12402040

[B53] Paxinos G, Watson C (2007) The rat brain in stereotaxic coordinates. London; Amsterdam: Elsevier.

[B54] Paz R, Bauer EP, Paré D (2008) Theta synchronizes the activity of medial prefrontal neurons during learning. Learn Mem 15:524–531. 10.1101/lm.932408 18612069 PMC2493046

[B55] Povysheva NV, Zaitsev AV, Rotaru DC, Gonzalez-Burgos G, Lewis DA, Krimer LS (2008) Parvalbumin-positive basket interneurons in monkey and rat prefrontal cortex. J Neurophysiol 100:2348–2360. 10.1152/jn.90396.2008 18632882 PMC2576192

[B56] Pratt WE, Mizumori SJ (2001) Neurons in rat medial prefrontal cortex show anticipatory rate changes to predictable differential rewards in a spatial memory task. Behav Brain Res 123:165–183. 10.1016/S0166-4328(01)00204-2 11399329

[B57] Preston AR, Eichenbaum H (2013) Interplay of hippocampus and prefrontal cortex in memory. Curr Biol 23:R764–R773. 10.1016/j.cub.2013.05.041 24028960 PMC3789138

[B58] Rajasethupathy P, Sankaran S, Marshel JH, Kim CK, Ferenczi E, Lee SY, Berndt A, Ramakrishnan C, Jaffe A, Lo M, Liston C, Deisseroth K (2015) Projections from neocortex mediate top-down control of memory retrieval. Nature 526:653–659. 10.1038/nature15389 26436451 PMC4825678

[B59] Rogan SC, Roth BL (2011) Remote control of neuronal signaling. Pharmacol Rev 63:291–315. 10.1124/pr.110.003020 21415127 PMC3082452

[B60] Ryou JW, Cho SY, Kim HT (2001) Lesions of the entorhinal cortex impair acquisition of hippocampal-dependent trace conditioning. Neurobiol Learn Mem 75:121–127. 10.1006/nlme.2000.3966 11222054

[B61] Sara SJ, Vankov A, Hervé A (1994) Locus coeruleus-evoked responses in behaving rats: a clue to the role of noradrenaline in memory. Brain Res Bull 35:457–465. 7859103 10.1016/0361-9230(94)90159-7

[B62] Schoenbaum G, Eichenbaum H (1995) Information coding in the rodent prefrontal cortex: I. Single-neuron activity in orbitofrontal cortex compared with that in pyriform cortex. J Neurophysiol 74:733–750. 10.1152/jn.1995.74.2.733 7472378

[B63] Solomon PR, Graves CA (1985) Classical conditioning of the nictitating membrane response in aged rabbits. Ann N Y Acad Sci 444:486–488. 3860105 10.1111/j.1749-6632.1985.tb37619.x

[B64] Squire LR (1992) Memory and the hippocampus: a synthesis from findings with rats, monkeys, and humans. Psychol Rev 99:195–231. 1594723 10.1037/0033-295x.99.2.195

[B65] Suh J, Rivest AJ, Nakashiba T, Tominaga T, Tonegawa S (2011) Entorhinal cortex layer III input to the hippocampus is crucial for temporal association memory. Science 334:1415–1420. 10.1126/science.1210125 22052975

[B66] Takehara-Nishiuchi K (2018) The anatomy and physiology of eyeblink classical conditioning. Curr Top Behav Neurosci 37:297–323. 10.1007/7854_2016_455 28025812

[B67] Takehara-Nishiuchi K, Kawahara S, Kirino Y (2005) NMDA receptor-dependent processes in the medial prefrontal cortex are important for acquisition and the early stage of consolidation during trace, but not delay eyeblink conditioning. Learn Mem 12:606–614. 10.1101/lm.5905 16322362 PMC1356179

[B68] Takehara-Nishiuchi K, McNaughton BL (2008) Spontaneous changes of neocortical code for associative memory during consolidation. Science 322:960–963. 10.1126/science.1161299 18988855

[B69] Takeuchi T, Duszkiewicz AJ, Sonneborn A, Spooner PA, Yamasaki M, Watanabe M, Smith CC, Fernández G, Deisseroth K, Greene RW, Morris RGM (2016) Locus coeruleus and dopaminergic consolidation of everyday memory. Nature 537:357–362. 10.1038/nature19325 27602521 PMC5161591

[B70] Tse D, Takeuchi T, Kakeyama M, Kajii Y, Okuno H, Tohyama C, Bito H, Morris RGM (2011) Schema-dependent gene activation and memory encoding in neocortex. Science 333:891–895. 10.1126/science.1205274 21737703

[B71] Tse MT, Piantadosi PT, Floresco SB (2015) Prefrontal cortical gamma-aminobutyric acid transmission and cognitive function: drawing links to schizophrenia from preclinical research. Biol Psychiatry 77:929–939. 10.1016/j.biopsych.2014.09.007 25442792

[B72] van Kesteren MTR, Rijpkema M, Ruiter DJ, Fernández G (2010) Retrieval of associative information congruent with prior knowledge is related to increased medial prefrontal activity and connectivity. J Neurosci 30:15888–15894. 10.1523/JNEUROSCI.2674-10.2010 21106827 PMC6633736

[B73] van Kesteren MTR, Ruiter DJ, Fernández G, Henson RN (2012) How schema and novelty augment memory formation. Trends Neurosci 35:211–219. 10.1016/j.tins.2012.02.001 22398180

[B74] Volle J, Yu X, Sun H, Tanninen SE, Insel N, Takehara-Nishiuchi K (2016) Enhancing prefrontal neuron activity enables associative learning of temporally disparate events. Cell Rep 15:2400–2410. 10.1016/j.celrep.2016.05.021 27264170

[B75] Wagatsuma A, Okuyama T, Sun C, Smith LM, Abe K, Tonegawa S (2018) Locus coeruleus input to hippocampal CA3 drives single-trial learning of a novel context. Proc Natl Acad Sci U S A 115:E310–E316. 10.1073/pnas.1714082115 29279390 PMC5777050

[B76] Wagner AD, Schacter DL, Rotte M, Koutstaal W, Maril A, Dale AM, Rosen BR, Buckner RL (1998) Building memories: remembering and forgetting of verbal experiences as predicted by brain activity. Science 281:1188–1191. 9712582 10.1126/science.281.5380.1188

[B77] Wang SH, Morris RGM (2010) Hippocampal-neocortical interactions in memory formation, consolidation, and reconsolidation. Annu Rev Psychol 61:49–79. 10.1146/annurev.psych.093008.100523 19575620

[B78] Weible AP, Weiss C, Disterhoft JF (2003) Activity profiles of single neurons in caudal anterior cingulate cortex during trace eyeblink conditioning in the rabbit. J Neurophysiol 90:599–612. 10.1152/jn.01097.2002 12750412

[B79] Wikenheiser AM, Schoenbaum G (2016) Over the river, through the woods: cognitive maps in the hippocampus and orbitofrontal cortex. Nat Rev Neurosci 17:513–523. 10.1038/nrn.2016.56 27256552 PMC5541258

[B80] Wilson RC, Takahashi YK, Schoenbaum G, Niv Y (2014) Orbitofrontal cortex as a cognitive map of task space. Neuron 81:267–279. 10.1016/j.neuron.2013.11.005 24462094 PMC4001869

[B81] Woodruff-Pak DS, Disterhoft JF (2008) Where is the trace in trace conditioning?. Trends Neurosci 31:105–112. 10.1016/j.tins.2007.11.006 18199490

[B82] Yau JO, McNally GP (2015) Pharmacogenetic excitation of dorsomedial prefrontal cortex restores fear prediction error. J Neurosci 35:74–83. 10.1523/JNEUROSCI.3777-14.2015 25568104 PMC6605253

[B83] Zhan C, Zhou J, Feng Q, Zhang JE, Lin S, Bao J, Wu P, Luo M (2013) Acute and long-term suppression of feeding behavior by POMC neurons in the brainstem and hypothalamus, respectively. J Neurosci 33:3624–3632. 10.1523/JNEUROSCI.2742-12.2013 23426689 PMC6619547

